# Poly(Ionic Liquid)s Dispersants for Lubricants: A Review on Structure–Property Relationships

**DOI:** 10.1021/acsomega.5c11446

**Published:** 2026-02-08

**Authors:** Nik Nur Azreen Nik Fauzi, JitKang Lim, Lauren Matthews, Ku Marsilla Ku Ishak, Mohamad Danial Shafiq

**Affiliations:** † School of Materials and Mineral Resources Engineering, Tuanku Syed Sirajuddin Engineering Campus, Universiti Sains Malaysia, 14300 Nibong Tebal, Penang, Malaysia; ‡ School of Chemical Engineering, Tuanku Syed Sirajuddin Engineering Campus, Universiti Sains Malaysia, 14300 Nibong Tebal, Penang, Malaysia; § ISIS Neutron and Muon Source, Rutherford Appleton Laboratory, Harwell Campus, Didcot, Oxfordshire OX11 0QX, U.K.

## Abstract

Poly­(ionic liquid)­s (PILs) have emerged as a versatile class of materials whose structural diversity, ranging from backbone chemistry and side-chain functionality to counterion type, governs their aggregation behavior, interfacial adsorption, and nanoscale self-assembly. These attributes make PILs attractive candidates for advanced applications in lubrication, dispersion stabilization, and sustainable functional materials. However, correlating molecular architecture with macroscopic performance remains a significant challenge, largely due to the complex and dynamic nature of their structural evolution. This review critically examines recent progress in understanding PIL structure–property relationships through multiscale characterization. Surface tension provides insight into interfacial activity, while small-angle X-ray scattering and small-angle neutron scattering elucidate nanoscale organization and hierarchical assembly. Complementarily, zeta-potential measurements quantify electrostatic interactions and the colloidal stability. Together, these techniques enable a coherent framework linking molecular design, interfacial phenomena, and bulk performance. Key knowledge gaps are highlighted, including the need for in situ and operando studies under realistic operating conditions as well as the integration of experimental techniques with computational modeling to capture dynamic restructuring and long-term stability. By synthesizing insights across structural chemistry, scattering techniques, and interfacial science, this review establishes future research directions for the rational design of PILs as next-generation dispersants, lubricants, and sustainable advanced materials.

## Graphical abstract

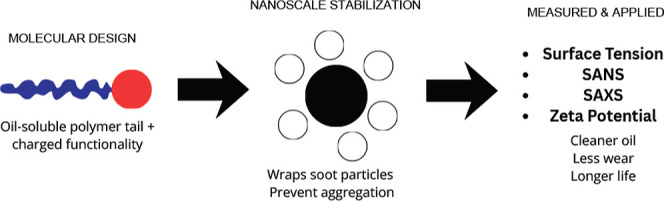

## Introduction

Automotive engines significantly contribute to environmental pollution, primarily through the emission of soot particles, which pose detrimental effects to both human health and the environment. Prolonged exposure to soot has been linked to respiratory diseases and, in severe cases, increasing risks of mortality.^1^ The formation of soot in combustion engines is a complex process influenced by the fuel composition, combustion temperature, pressure, and oxygen availability. Due to the high surface reactivity of soot particles, these micro/nanoparticles can form aggregates and eventually large agglomerates that contribute to engine wear and operational inefficiency.^2^ Lubrication plays a critical role in mitigating friction and wear in mechanical systems, ensuring efficient performance and extending the lifespan of mechanical components.^3^ Engine lubricants are typically composed of base oils combined with performance-enhancing additives, such as dispersants. Dispersants maintain the suspension state of contaminants, including soot and sludge, preventing their agglomeration and deposition on engine surfaces.^4^ Polyisobutylene succinic anhydride (PIBSA)-based dispersants have been widely employed due to their compatibility with lubrication systems and their ability to stabilize particulate matter. Among the various polyisobutylene succinic anhydride (PIBSA)-derived dispersants, polyisobutylene succinimide (PIBSI) has emerged as one of the most extensively utilized, primarily due to its effective dispersity and thermal stability. PIBSI is typically synthesized by condensing PIBSA with polyamines, forming imide linkages within the molecular structure.^5^ While conventional engine oil formulations predominantly incorporate dispersants based on a polyisobutylene–polyamine system, the molecular architecture, particularly the nature of the intermediate linkages and subsequent chemical modifications, is often engineered to optimize performance characteristics, such as thermal oxidation resistance, soot dispersion, and compatibility with other additive components within the lubricant system.

The imide functional group exhibits a strong affinity for the soot particle surface, which typically contains oxygenated functional groups, such as hydroxyl, carboxyl, and carbonyl, introduced during the combustion cycle or oxidative aging of the lubricant. These surface functionalities enable hydrogen-bonding and acid–base interactions with the imide headgroups, thereby facilitating robust adsorption of PIBSI onto soot particles.^6^ Once anchored, the solvated PIB chains extend into the oil medium, creating a steric barrier that effectively prevents the close approach and subsequent agglomeration of soot particles.^7^ This steric hindrance is essential in low-polarity lubricating oils, where electrostatic stabilization is negligible due to long-range interactions among carbon particles. The dispersibility of soot in the oil matrix is further enhanced by the oleophilic PIB backbone, which improves the compatibility of the soot-PIBSI complex within the lubricant system. It has been reported that the molecular weight of the PIB segment, the architecture of the dispersant, and the surface chemistry of soot particles are critical parameters influencing the effectiveness of soot stabilization.^8^

While PIBSI dispersants are widely applied in the modern engine lubrication technology due to their proven effectiveness, some inherent performance trade-offs may affect long-term durability and formulation flexibility under demanding conditions. At the elevated operating temperatures characteristic of internal combustion engines, dispersants perform a vital function yet are subjected to thermal and oxidative conditions that can affect their long-term chemical stability, potentially reducing dispersibility efficiency and increasing carbonaceous deposits.^9^ Furthermore, like any other traditional dispersants, PIBSI exhibit a finite soot-carrying capacity; once the soot concentration exceeds the critical threshold, particle agglomeration becomes inevitable, potentially resulting in oil thickening, filter plugging, and, eventually, accelerated engine wear.^10^ The efficiency of soot stabilization by PIBSI is also highly dependent on its molecular architecture, particularly the polyamine structure, the polyisobutylene (PIB) chain length, and the density of active adsorption sites.^6^ Improper molecular design can significantly compromise the steric stabilization and adsorption efficiency. PIB-based dispersants, such as PIBSI, may exhibit compatibility constraints with certain lubricant additives, including detergents and friction modifiers; this behavior can be further influenced by suboptimal additive formulation.^11^

Recent research has revealed ambiguities in the mechanisms involved and performance limitations of PIBSI dispersants, challenging the commonly accepted understanding of their soot-stabilization behavior in oil. Simulation-based studies have demonstrated that PIBSI–soot interactions arise from the combined influence of steric, entropic, and interfacial effects rather than steric repulsion alone.^12^ Under high soot loading, restricted PIB chain mobility may lead to premature agglomeration despite an apparently adequate dispersant concentration.^5^ This inconsistency highlights the limitations of soot-carrying capacity predictions, which rely exclusively on steric stabilization. Besides, advanced oxidation studies have also revealed that at high temperatures and high-shear regimes, dispersant molecules undergo competitive pathways of radical-driven scission and cross-linking, forming unstable intermediates, contributing to sludge formation, which leads to reduced effective dispersancy over time.^13^

Such oxidative degradation pathways are yet to be fully incorporated into classical models describing the dispersant’s performance, highlighting essential gaps in current understanding. Recent work on polymeric dispersants further underscores that subtle changes in molecular architecture significantly affect soot–particle interactions, adsorption behavior, and micelle-like structuring in oil, implying that the dispersant’s performance is more sensitive to structural parameters than previously considered. These emerging insights collectively underscore that real-engine conditions are far more complex and less predictable than classical models suggest for PIBSI and other traditional dispersants behavior. Thus, an evolving understanding reinforces motivation to explore polymeric ionic liquids (PILs), which offer tunable ionic architectures, robust interfacial interactions, enhanced oxidative resistance, along with multifunctional stabilizing mechanisms beyond what could be achieved in the classical dispersants.^14^

These PIBSI and other conventional dispersant limitations emphasize the need for dispersants that overcome steric-only stabilization constraints, with PILs offering notable advantages.^15,16^ IL segment of PILs feature tunable cation–anion pairs that enable strong electrostatic interactions, hydrogen bonding, and π–π stacking with soot surfaces, providing stabilization mechanisms inaccessible through steric hindrance alone.^17^ The ionic nature enhances interfacial affinity, promoting efficient adsorption and improved soot dispersion even under high soot loading, complementing the strong baseline performance of PIBSI, which remains effective under typical engine conditions. PILs also exhibit superior oxidative resistance^18^ and are less prone to radical-driven degradation compared to the oxidation-induced breakdown commonly exhibited by PIBSI during severe engine operation conditions.^19^ Within low-SAPS (sulfurated ash, phosphorus, and sulfur) lubricant formulations, conventional dispersants continue to deliver effective soot stabilization under typical engine operating conditions, maintaining reliable engine protection despite reduced detergent synergy. Complementing this, the intrinsic polarity and multifunctional nature of PIL structures enable sustained dispersancy under challenging conditions, such as high soot loading, extreme temperatures and limited coadditives, offering additional stabilization mechanisms that extend beyond those accessible to conventional dispersants.^20^ These attributes highlight the ability of PILs to overcome several of the inherent weaknesses of conventional dispersants, providing a strong rationale for their exploration as next-generation soot-control additives.

To address these limitations, ILs have emerged as promising alternative dispersants and multifunctional lubricant additives. ILs are composed entirely of ions and exhibit superior thermal stability, negligible volatility, and highly tunable chemical structures. These characteristics enable their customization for enhanced soot dispersion, improved surface adsorption, and synergistic interactions with other lubricant additives.^21^ Typically, ILs consist of bulky organic cations paired with either organic or inorganic anions and remain in the liquid state at temperatures below 100 °C.^22^ Their inherent polarity, adjustable solubility, and exceptional thermal resilience position ILs as highly promising candidates for next-generation lubricant formulations. Studies have shown ILs, particularly those incorporating functionalized phosphonium or imidazolium cations with appropriately selected anions, can adsorb efficiently onto soot surfaces, providing both steric and electrostatic stabilization in nonpolar media.^23,24^ ILs also exhibit excellent oxidative stability, high thermal resistance, and strong compatibility with a wide range of lubricant base liquids. Their unique molecular architecture and multifunctional properties enable ILs as promising alternatives, and synergistic coadditives to conventional PIBSI dispersants in the formulation of next-generation lubricants for high-efficiency, low-emission engines.^25^

While many studies have reported on IL lubrication, the discussion has predominantly centered on ILs functions as friction modifiers, antiwear additives, and neat lubricants. These works emphasize tribological performance but largely overlook recent developments in PIBSA-derived, oil-soluble ILs and PILs that operate through complex interfacial and colloidal mechanisms. Moreover, current reviews provide limited insight into how ionic architecture, counterion selection, alkyl chain functionality, or polymer grafting strategies influence soot stabilization and dispersant performance in engine oils. Most significantly, the existing literature lacks a systematized, multiscale framework that links PIL synthesis routes, particularly those derived from PIBSA chemistries, to nanoscale structure and macroscopic dispersancy performance. Recent advances in structural and surface characterization techniques have enabled detailed molecular- and interfacial-level insights into IL and PIL aggregation behavior, adsorption at oil–solid interfaces, and interactions with soot particles. However, these emerging insights into structure–interaction relationships and aggregation processes are yet to be systematically integrated or critically synthesized in any prior review.

This review presents a comprehensive and novel perspective by integrating molecular design principles, structural characterization, and dispersant- and tribological–relevant interaction mechanisms. Accordingly, IL and PIL systems are placed within a more clearly defined scientific framework, highlighting their potential as new-generation dispersants that can overcome the mechanistic limitations of PIBSI-based additives. Specifically, this review also presents a comprehensive analysis of oil-soluble ILs for dispersant development with emphasis on their interactions with PIBSA, the structural and functional attributes of phosphonium-based PIL dispersants, and the characterization techniques used to elucidate these relationships. By consolidating recent advances and emerging insights, this review aims to support the development of high-performance and more sustainable lubricant technologies.

### Oil-Soluble ILs and PIBSA Reaction

#### Characteristics of Oil-Soluble ILs

ILs are a class of environmentally friendly solvents composed entirely of ions, typically consisting of large, asymmetric organic cations and smaller inorganic or organic anions, and they exist as liquids at or near room temperature.^26^ This behavior arises from poor lattice packing and weakened Coulombic interactions, resulting in low melting points, with most ILs defined as salts that melt below 100 °C.^21^ ILs were originally developed for electrochemical applications, such as electrolytes in batteries and for electrodeposition. However, they have since emerged as “green” solvents due to their negligible vapor pressure, high thermal and chemical stability, and excellent solvating power across a wide range of solutes.^27^ For instance, the widely studied 1-butyl-3-methylimidazolium hexafluorophosphate ([BMIM]­[PF_6_]) and trihexyl­(tetradecyl)­phosphonium bis­(2,4,4-trimethylpentyl)­phosphinate ([P_66614_]­[BTMPP]) have demonstrated exceptional thermal stability and nonvolatility, enabling their use in harsh environments.^28,29^ Their low volatility minimizes emissions and flammability risks, and their tunable molecular structure enables precise control of properties, such as viscosity, polarity, and miscibility. These distinctive properties allow ILs to serve as tunable, task-specific solvents and functional additives.

One of the most significant advantages of ILs is their tunable solubility in both polar and nonpolar environments, a feature that critically depends on their molecular design. In hydrophobic media such as hydrocarbons or base oils, IL solubility is primarily influenced by the cation’s alkyl chain length, the presence of nonpolar substituents, and the hydrophobicity of the anion.^30^ For example, [P_66614_]­[NTf_2_] (trihexyl­(tetradecyl)­phosphonium bis­(trifluoromethylsulfonyl)­imide) exhibits complete miscibility with polyalphaolefin (PAO) base oils due to its long hydrocarbon chains and fluorinated anion, which enhances van der Waals compatibility with apolar solvents.^31^ Similarly, 1-octyl-3-methylimidazolium triflate ([OMIM]­[TfO]) shows improved oil miscibility compared to its shorter-chain substitutes, making it suitable for oil-based dispersant systems. In contrast, more polar ILs such as [BMIM]­[Cl] and [EMIM]­[BF_4_] are largely immiscible with nonpolar media due to their strong ionic character and lack of hydrophobic groups.^32^

These molecularly engineered ILs have become increasingly relevant in petroleum-related applications including extraction, lubrication, and catalysis in hydrocarbon-rich environments. Their inherent thermal resilience, nonflammability, and ability to operate under high-temperature and high-pressure conditions further emphasize their suitability as functional additives in advanced lubricant formulations. [P_66614_]­[BTMPP] has been studied for use in engine oils due to its enhanced tribological performance and high thermal oxidative stability, forming durable antiwear films on metal surfaces.^33^ Similarly, ILs based on phosphonium cations and sulfonate or phosphate anions have demonstrated multifunctional behavior as antiwear agents, friction modifiers, and dispersants, outperforming conventional additives like zinc dialkyldithiophosphate (ZDDP) in some cases.^34^

Common IL cations include imidazolium, phosphonium, ammonium, pyridinium, pyrrolidinium, and guanidinium derivatives. These cations are typically large and asymmetric, often featuring alkyl substituents (R groups) that can be varied to modulate polarity, viscosity, and solubility.^35^ The anionic components are even more diverse and include both inorganic anions such as hexafluorophosphate (PF_6_^–^), tetrafluoroborate (BF_4_^–^), triflate (TfO^–^), and chloride (Cl^–^), as well as organic anions like dicyanamide (DCA^–^), alkyl sulfates, mesylate, and bis­(trifluoromethylsulfonyl)­imide (NTf_2_^–^), as depicted in Figure 1.^22^ Additionally, more structurally complex anions such as bis­(2,4,4-trimethylpentyl)­phosphinate (BTMPP^–^) offer further opportunities to tailor the IL behavior. For example, BTMPP^–^-based ILs exhibit high oil solubility and low polarity, making them ideal for use in hydrocarbon-rich systems, such as lubricant formulations or wax-dispersion media.^36^

**Figure 1:**
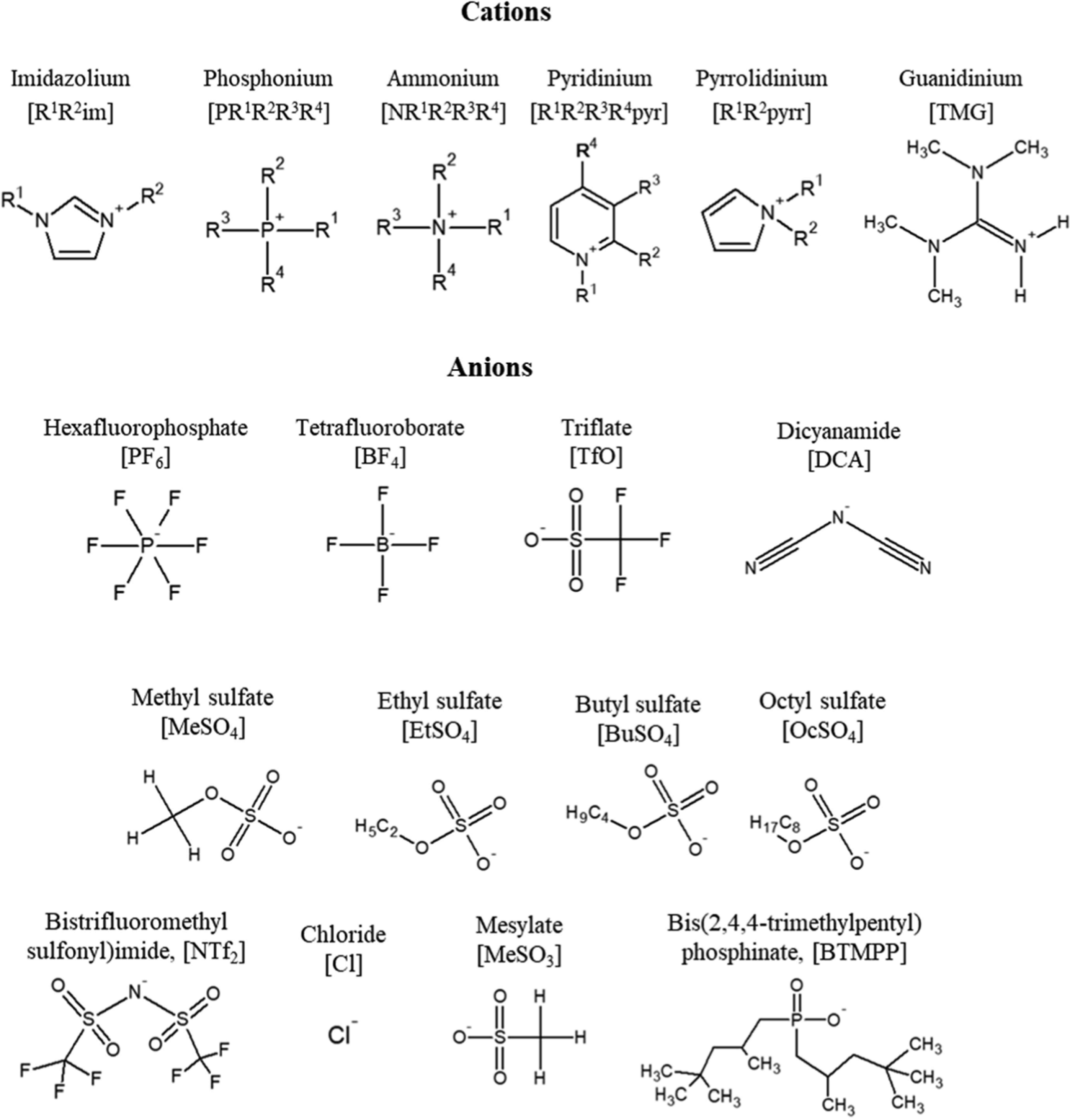
Structures of the cations and anions. Adapted with permission from [ref 22]. Copyright [2021]­[Elsevier].

ILs are immiscible with nonpolar molecules due to fundamental differences in intermolecular interactions: ions associate primarily through strong Coulombic forces and, in some cases, hydrogen bonding, whereas nonpolar molecules interact via relatively weak van der Waals forces.^37,38^ Oil–IL miscibility is typically assessed by visual inspection, in which phase separation or turbidity indicates immiscibility; this is followed by centrifugation to confirm phase boundaries, exploiting the higher density of ILs relative to hydrocarbon oils.^39,40^ Thermal methods, such as heating or freezing the mixture, can be employed to assess further the thermodynamic stability of the oil–IL system.^41^ Some studies also report the use of dynamic light scattering spectroscopy and Nuclear Magnetic Resonance (NMR) diffusometry to quantify IL dispersion stability in oil matrices.^42^

#### Reaction with PIBSA

Polyisobutylene succinic anhydride (PIBSA) is a pivotal intermediate in the synthesis of dispersants engineered to meet the performance demands of modern, energy-efficient, and environmentally responsible engine oils. Its extensive adoption in lubricant formulations stems from its robust ability to anchor onto soot and sludge particles via polar succinimide functionalities while maintaining solubility in nonpolar oil matrices, thus providing robust dispersancy.^43^ PIBSA acts as a multifunctional platform molecule, serving as a precursor for dispersants and emulsifiers and imparting supplementary benefits such as antiwear performance, corrosion inhibition, viscosity index improvement, and enhanced lubricity in both engine oils and fuel systems.^25^ Recent advancements in the design of lubricant additives have focused on the integration of oil-soluble ILs into PIBSA frameworks to synthesize next-generation dispersants with tunable chemical functionality and excellent performance attributes.^44^ The succinic anhydride moiety in PIBSA readily undergoes nucleophilic ring-opening reactions with reactive functional groups present in ILs, most notably primary or secondary amines, hydroxyls, and phosphonium derivatives, leading to the formation of imide, amide, ester, or ionically tethered derivatives.^45^ These reactions yield polymeric ionic liquid (PIL) dispersants that exhibit enhanced amphiphilicity, enabling the improved dispersion of soot and sludge in hydrocarbon-based lubricant systems.

Structurally diverse ILs have been employed in this context. For example, functionalized imidazolium-based ILs such as 1-aminopropyl-3-methylimidazolium bromide ([APMIm]­[Br]) and *N*-butyl-*N*-methylpyrrolidinium bis­(trifluoromethylsulfonyl)­imide [Pyr_14_]­[TFSI] have been shown to react efficiently with PIBSA to form PILs with stable imide linkages.^46,47^ These materials exhibit significant improvements in thermal and oxidative stability, often withstanding degradation at temperatures exceeding 300 °C, as confirmed by thermogravimetric analysis (TGA) and pressure differential scanning calorimetry.^48^ Moreover, phosphonium-based ILs, such as trihexyl­(tetradecyl)­phosphonium bis­(2,4,4-trimethylpentyl)­phosphinate ([P_66614_]­[BTMPP]), have demonstrated exceptional oil solubility due to their long alkyl chains and bulky, hydrophobic anions, making them highly suitable for functionalization of PIBSA in Group III and polyalphaolefin (PAO) base oils.^49^

These PIL-modified dispersants exhibit a synergistic combination of steric and electrostatic stabilization mechanisms. The polyisobutylene backbone confers steric hindrance to suppress agglomeration, while the ionic moieties derived from ILs introduce electrostatic repulsion, thereby enhancing soot dispersibility under severe lubrication conditions. Compared with conventional PIBSI systems, PIL-based dispersants display excellent performance in sludge dispersion, high-temperature shear stability, and resistance to oxidative breakdown. Such advancements align with current regulatory and performance-driven demands for thermally robust, low-ash, and environmentally compliant lubricant formulations, positioning PILs as a promising class of multifunctional additives for next-generation engine oil technologies.^25,50^ The key chemistries and resulting structures of the IL-PIBSA reactions, along with their functional implications in lubrication, are summarized in Table 1.

**Table 1:** Reaction of ILs with PIBSA

IL structure	reaction with PIBSA	chemical groups involved	oil solubility
imidazolium-based ILs	polar imidazolium-based ILs may react with the polar succinic anhydride present in PIBSA, yielding a stabilized oil-soluble dispersant	low polarity of imidazolium ring, –NH, –COOH groups offers reactivity	less soluble in oil than other ionic liquids, but can be improved by attaching longer alkyl chains^53^
	hydrophilicity can be imparted through the polar imidazolium group and is further considered to serve as a site for hydrogen bonding or electrostatic interactions5 ^51^	the polarity of imidazolium-based ILs depends on both cation alkyl chain length and anion coordinating strength; ILs with longer alkyl chains and weakly coordinating anions such as NTf_2_^–^ or PF_6_^–^ exhibit lower polarity	
		the imidazole ring is a five-membered heterocyclic structure containing two nonadjacent nitrogen atoms. Quaternization of this ring generates a positively charged imidazolium cation5 ^52^	
phosphonium-based ILs	electrostatic attraction between the positively charged phosphonium cation of the ionic liquid and negatively charged carbonyl oxygen atoms of the PIBSA anhydride group^54^	cationic; the phosphonium cation [PR_4_]^+^ is the central part of the ionic liquid and is composed of a phosphorus atom surrounded by four organic groups (R) which can be alkyl chains^55^	highly soluble in oil, especially if designed with long alkyl chains on the phosphonium cation, making them suitable as additives in lubricants due to their good miscibility with oil-based fluids^56,57^
	the dispersion effect that concerning the alkyl chains attached to the phosphonium cation, ionic liquid may also act as a surfactant to aid PIBSA molecules dispersion within a nonpolar medium by creating a micelle-like structure^46^		
ammonium-based ILs	ionic interaction with polar regions within the molecules	quaternary ammonium ion-four butyl groups, where all of these have been substituted with alkyl groups to provide a positively charged cation and serves as a precursor for forming ionic liquid along with an anion^59^	different kinds of ammonium-based ionic liquids have a strong affinity to dissolve in oils, especially when the cations contain long alkyl chains. Thus, ILs can serve as additives to oils in the application of lubrication or treatment of heavy oil^60^
	reaction that results in the formation of an amide or imide by the nucleophilic attack of the anhydride group by ammonium nitrogen^58^		
pyrrolidinium-based ILs	polar groups of the pyrrolidinium cation interact with PIBSA anhydride or form ionic bonds	belongs to the chemical grouping of “cyclic ammonium cations” where the positively charged ion is derived from a pyrrolidine ring, a five-membered cyclic amine, with an alkyl group attached to the nitrogen atom, making it a quaternary ammonium compound^61^	pyrrolidinium-based ILs can be made to be oil-soluble, as long chains attached to the pyrrolidinium cation allow them to interact favorably with nonpolar hydrocarbon molecules in oil; hence, they are more compatible and soluble within the oil phase^62^
guanidinium-based ILs	forming ionic bonding with PIBSA or reacting covalently by nucleophilic attack	It is considered to be a family of chemicals, in which the cation is a guanidium ion, a positively charged nitrogen group with the chemical formula C(NH_2_)_3_^+^, bearing different alkyl chains on each nitrogen, and can thus be varied. This cation is then joined with a negatively charged anion to produce an ionic liquid^63^	In general, these salts have shown a good solubility in oils if properly designed by means of suitable alkyl chains at the guanidinium cation that interact with the hydrophobic portions of the oil molecules^64^

### Reaction Efficiency

The functionalization of PIBSA with ILs represents a promising strategy to suppress carbon sludge formation in lubricant formulations. This reaction leverages the unique physicochemical properties of ILs, particularly their polarity, ionic conductivity, and molecular tunability in order to enhance the solubilization and dispersion of sludge precursors such as oxidized hydrocarbons, soot, and resinous byproducts. When grafted onto PIBSA, ILs not only improve the amphiphilic balance of the dispersant but also facilitate more effective interactions with polar contaminants, thereby improving the colloidal stability and reducing sludge agglomeration in oil matrices.

ILs can function as solvents, cosolvents, or reactive additives during the grafting process. The reaction typically proceeds via a nucleophilic ring-opening mechanism of the succinic anhydride group in PIBSA, often under elevated temperatures to ensure sufficient reactivity and mixing. Reaction temperatures between 60 and 150 °C are commonly employed, consistent with those used in analogous polymer–solvent systems.^65^ Depending on the polarity and solubility parameters of the IL and PIBSA precursors, ILs themselves may serve as the reaction medium. However, in cases where mutual solubility is limited, aprotic solvents such as toluene, xylene, decane, or dichloromethane are often employed to ensure homogeneous dispersion and favorable reaction kinetics.^65^

Furthermore, the molar ratio of IL to PIBSA must be carefully optimized to maximize the performance without compromising thermal or oxidative stability. Excess IL may lead to phase instability or unreacted residues, whereas insufficient IL loading may limit the functional group conversion. Characterization studies, including Fourier-transform infrared spectroscopy (FTIR), thermogravimetric analysis (TGA), NMR, and elemental analysis, have confirmed the successful formation of ionic or covalent PIBSA–IL adducts. These materials demonstrate more effective performance in dispersancy, thermal robustness, and sludge inhibition compared to conventional polyisobutylene succinimide (PIBSI) counterparts.^4,66^

Pandey and co-workers (2021)^24^ employed FTIR and proton nuclear magnetic resonance (^1^H NMR) to confirm the efficient interaction between ILs and PIBSA, leading to the formation of polyisobutylene succinimide-based ionic liquid (PIBIL) dispersants. Specifically, the phosphonium-based IL trihexyl­(tetradecyl)­phosphonium decanoate ([P_66614_]­[Dec]) was examined for its interaction with PIBSA and its subsequent reaction product, PIBIL, across varying concentrations and temperatures. Spectroscopic analyses revealed characteristic shifts in chemical environments indicative of ring-opening reactions and the formation of covalent or ionic adducts. The ^1^H NMR spectra showed changes in the chemical shifts of methylene and alkyl protons, while FTIR spectra confirmed the consumption of anhydride functional groups and the emergence of imide or ester bands, validating the structural modification of PIBSA by the IL.

Figure 2 shows broadening and marginal shift in the anion region of the P_66614_ Dec, which suggest the interaction between PIBSA and the P_66614_ Dec blend. As the concentration of PIBSA increases, there was broadening and diminishing of the decanoate peak at 1577 cm^–1^ of the IL, which suggests the interaction and encapsulation of the IL by PIBSA. The encapsulation restricts the cation and anion of the IL, which come in close proximity to each other. At 40% PIBSA-60% IL, there was no carboxylate peak for decanoate observed, which suggests the fully encapsulated P_66614_ Dec by PIBSA through noncovalent interactions such as ion-pair, dipole-ion, and dipole–dipole interactions.^25^

**Figure 2:**
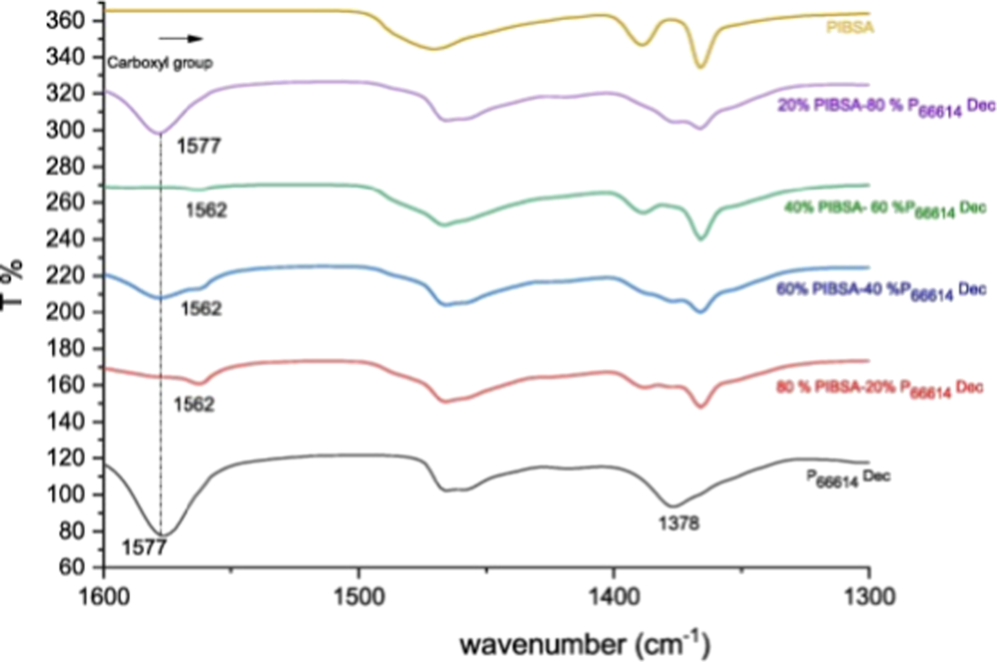
FTIR spectrum for P_66614_ Dec with the concentration variation of PIBSA. Adapted with permission from [ref 25]. Copyright [2021]­[Elsevier].

Figure 3 presents the temperature-dependent ^1^H NMR behavior of P_66614_ Dec over the range −50 to 50 °C, examined in its neat form and in the presence of equimolar additions of the IL with the dispersants PIBSA and the conventionally used polyisobutylene succinimide (PIBSI). For neat P_66614_ Dec, the chemical shifts remained largely unchanged across the investigated temperature range. Upon increasing the temperature to 50 °C, a slight downfield shift of the cationic resonances is observed, whereas cooling to subambient temperatures results in a minor upfield shift. These temperature-induced variations are attributed to changes in the average donor–acceptor separation arising from reduced intermolecular interactions at elevated temperatures. Notably, the temperature dependence of the interactions between the IL and the PIBSA and PIBSI additives differs markedly, indicating distinct interaction mechanisms for the two dispersants. As the temperature was reduced from 50 to −50 °C, it was observed that peaks merged when PIBSA was blended with P_66614_ Dec. This is comparable to the concentration variation of PIBSA and IL. This suggests that PIBSA restricts the movement and entraps the IL due to interactions like ion pairing, hydrogen bonding, and ion–dipole. The merging of the cationic and anionic resonances at −4 °C suggests that the IL experiences a common local environment, indicative of close cation–anion proximity arising from molecular interactions with PIBSA. To further elucidate the nature of these interactions between the dispersant and the P_66614_ Dec IL, the dispersant was subsequently replaced with PIBSI, a chemically analogous additive differing primarily in the headgroup chemistry, where the anhydride functionality in PIBSA is substituted by an imide group. As no discernible changes in peak positions were observed, the addition of PIBSI was found to have a negligible effect on the ion-association behavior of the IL, in contrast to the pronounced interactions observed with PIBSA. This difference may be attributed to the absence of a free NH_2_ functionality in PIBSI (bis-succinimide), which reduces the availability of interaction sites, such as hydrogen-bond donors or dipole–dipole interaction centers, thereby limiting its ability to interact with P_66614_ Dec.

**Figure 3:**
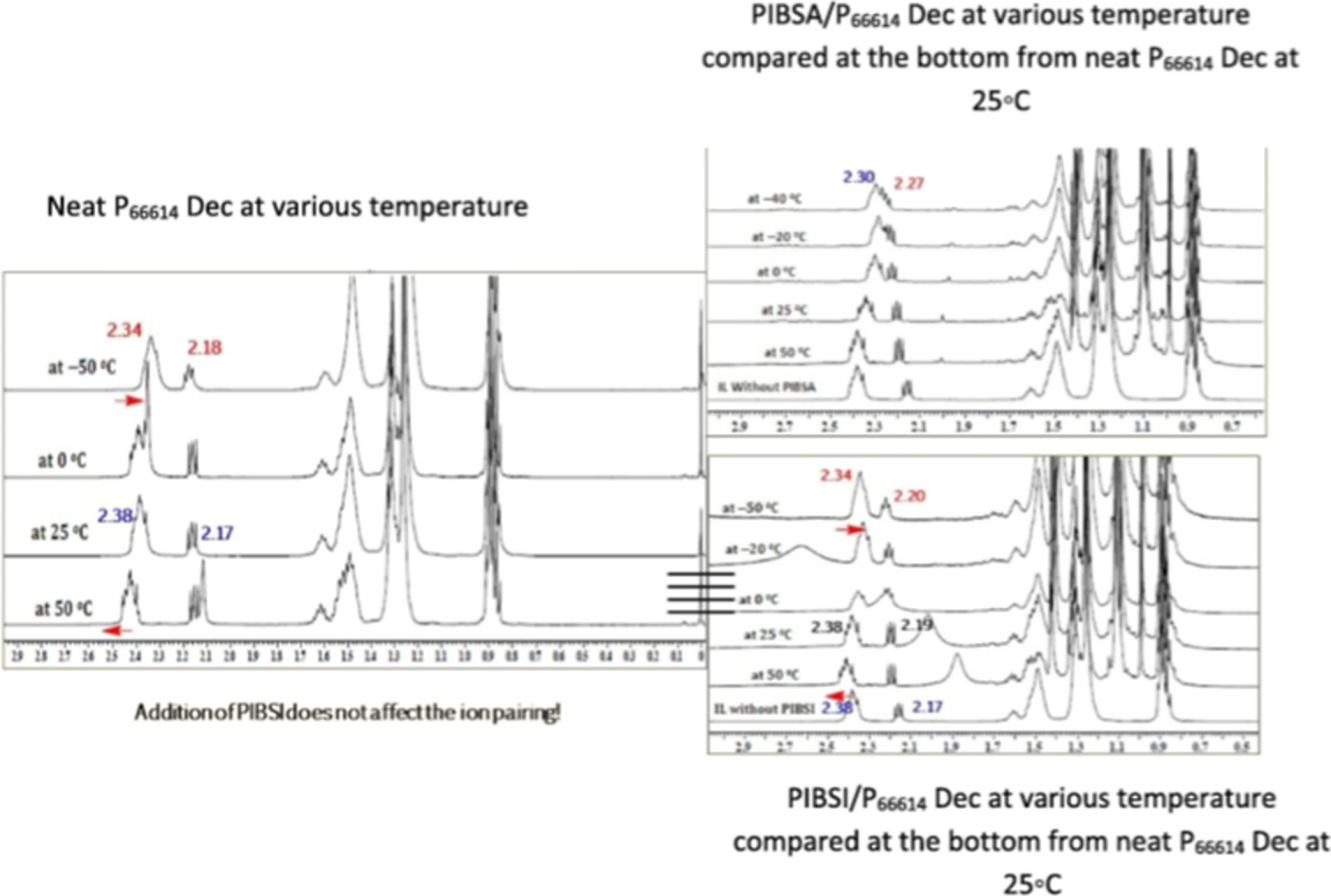
^1^H NMR shifts on the left-hand side shows influence of temperature on neat P_66614_ Dec IL and right-hand side shows equimolar mixtures of PIBSA/PIBSI—P_66614_ Dec lubricant IL at various temperatures which was compared at the bottom from neat P_66614_ Dec IL. Adapted with permission from [ref 25]. Copyright [2021]­[Elsevier].

In addition, Zhang and co-workers (2017)^46^ reported the design and synthesis of a halogen-free, boron-containing polyisobutylene-based IL (PIBIL) that exhibits excellent compatibility with Group I–IV hydrocarbon base oils as well as formulated engine oils. Notably, incorporation of PIBIL resulted in significant improvements in the antiwear performance. Figure 4 illustrates the synthesis of the PIBIL lubricant additive, including the preparation of lithium bis­(salicylato)­borate (LiBScB), 1-aminopropyl-3-methylimidazolium bromide ([APMIm]­[Br]), 1-aminopropyl-3-methylimidazolium bis­(salicylato)­borate ([APMIm]­[BScB]), and the final PIBIL product. Structural confirmation by ^1^H NMR and ATR spectroscopy verifies the successful chemical conversion of PIBSA to a PIL rather than a physical blend.

**Figure 4:**
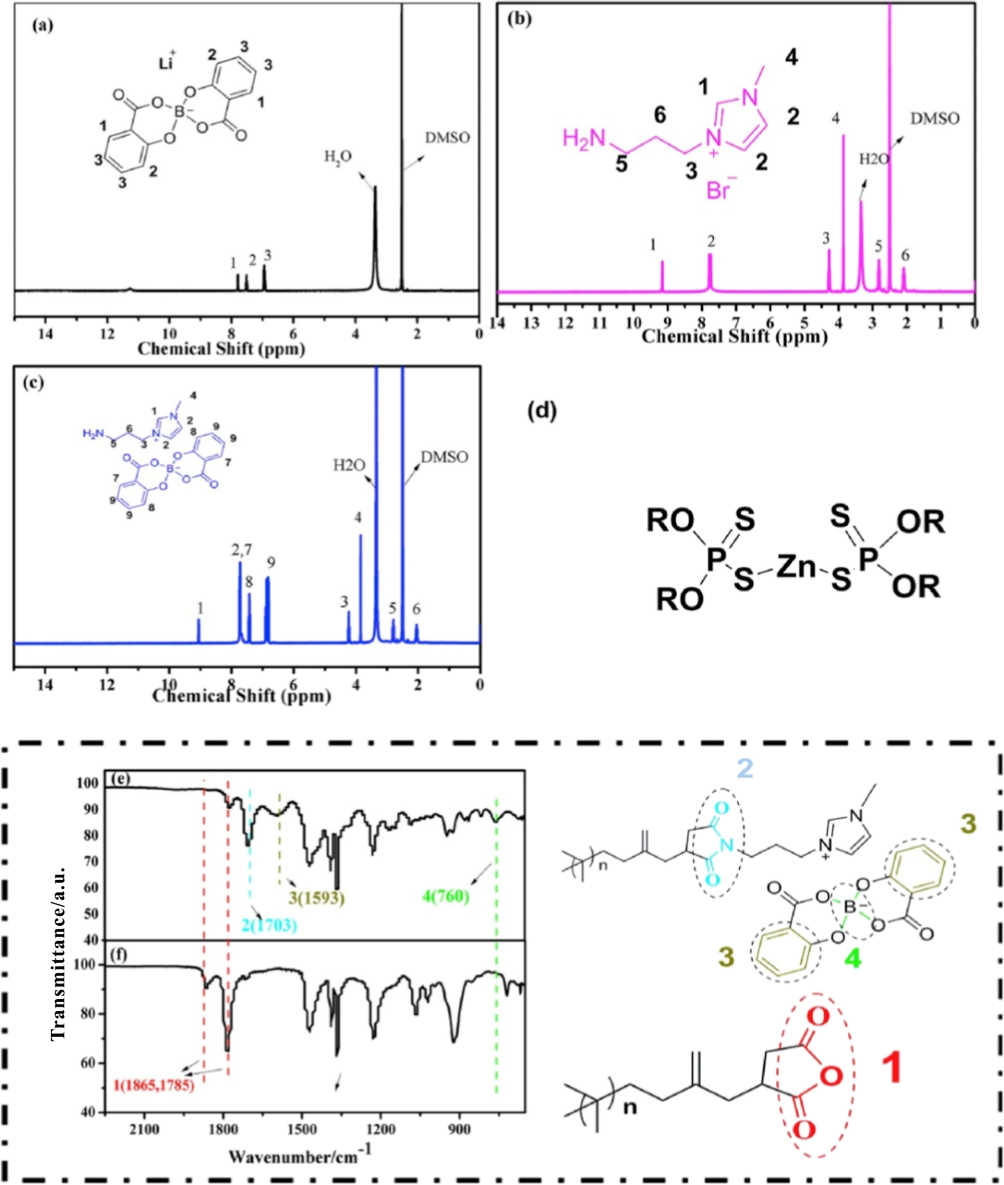
Structure of ^1^H NMR of LiBScB (a), [APMIm] [Br] (b), [APMIm] [BScB] (c); structure of ZDDP (d); structures and ATR of PIBIL (e), PIBSA (f). Adapted with permission from [ref 46]. Copyright [2017]­[Elsevier].

Based on this work, PIBIL was shown to significantly enhance antiwear performance in Group I–IV hydrocarbon base oils and engine oils through comprehensive tribological evaluation, thermal analysis, surface characterization, and oil-solubility assessment. Figure 5 presents the thermogravimetric analysis/derivative thermogram (TGA/DTG) comparison between PIBIL and the conventional zinc dialkyldithiophosphate (ZDDP). ZDDP begins to decompose at a lower temperature (≈ 185 °C), whereas PIBIL exhibits substantially higher thermal stability, with the maximum rate of mass loss occurring at around 400 °C. In addition, due to its zinc content, ZDDP leaves approximately 20% solid residue after decomposition, while PIBIL undergoes continuous mass loss and leaves almost no ash, confirming its low-ash nature.

**Figure 5:**
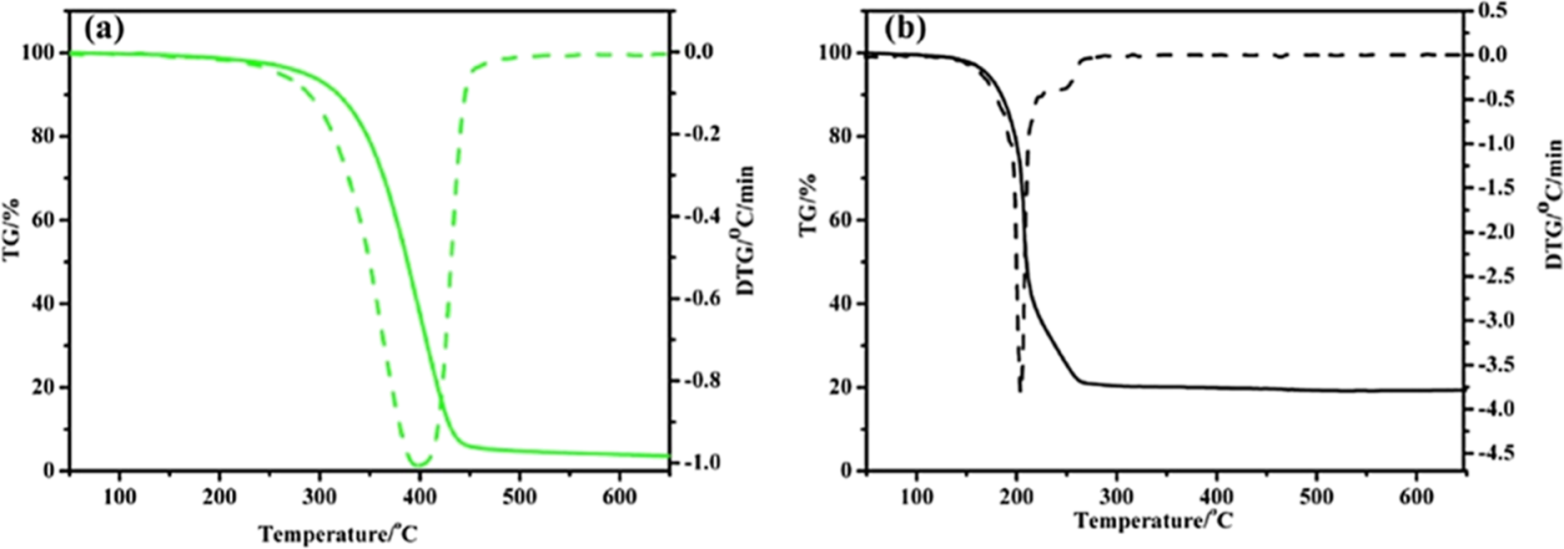
TGA/DTG curves of PIBIL (a) and ZDPP (b). Adapted with permission from [ref 46]. Copyright [2017]­[Elsevier].

Zhang and co-workers (2017)^46^ also evaluated the tribological behavior of PIBIL in base oil using a four-ball tester. When used as a sole additive, PIBIL exhibited concentration-dependent friction behavior, with inferior performance at low concentrations attributed to competitive mechanical stress interactions between PIBIL molecules and the base oil. Nevertheless, wear was consistently reduced compared with neat base oil, indicating that PIBIL possesses intrinsic antiwear capability. When PIBIL was combined with ZDDP at a fixed total additive concentration of 1 wt %, all binary formulations showed a marked reduction in wear relative to the base oil. The optimal formulation was observed at 0.50 wt % PIBIL and 0.50 wt % ZDDP, which produced the greatest reductions in both the coefficient of friction and wear scar diameter, demonstrating a pronounced synergistic effect. Microscopic examination and elemental analysis of worn steel surfaces supported these findings. Compared with neat base oil and ZDDP-only lubrication, the use of PIBIL resulted in shallower grooves and reduced surface damage, while the combined PIBIL–ZDDP formulation produced the smallest wear scars and effectively eliminated scuffing. Elemental analysis confirmed the presence of zinc, phosphorus, sulfur, boron, and related species on the contact interface, indicating additive-derived material transfer during sliding.

X-ray photoelectron spectroscopy analysis revealed that lubrication with PIBIL alone resulted in the formation of boron- and nitrogen-containing species on the worn surface, consistent with delayed decomposition arising from the higher thermal stability of PIBIL. In the presence of both PIBIL and ZDDP, boron-, phosphorus-, oxygen-, and nitrogen-containing compounds were simultaneously detected, confirming the formation of a compact and chemically stable hybrid tribofilm generated through tribochemical interactions between the two additives.^46^

The time-dependent evolution of the coefficient of friction further elucidates the synergistic mechanism. Figure 6 shows that PIBIL reaches a stable friction state rapidly, within approximately 90 s, indicating fast film incubation due to its polar ionic moieties. In contrast, ZDDP requires a longer induction period (≈ 380 s) before reaching a steady state. Although the tribofilm formed by PIBIL alone may undergo partial disruption during prolonged sliding, the binary PIBIL–ZDDP formulation exhibits a stable friction throughout the test duration. This behavior suggests that PIBIL promotes rapid surface adsorption, while ZDDP subsequently decomposes and reacts to form a durable hybrid boundary film. The interaction between amine-based PIBIL and ZDDP, schematically illustrated in Figure 6b, is therefore crucial to the observed synergistic antiwear performance.

**Figure 6:**
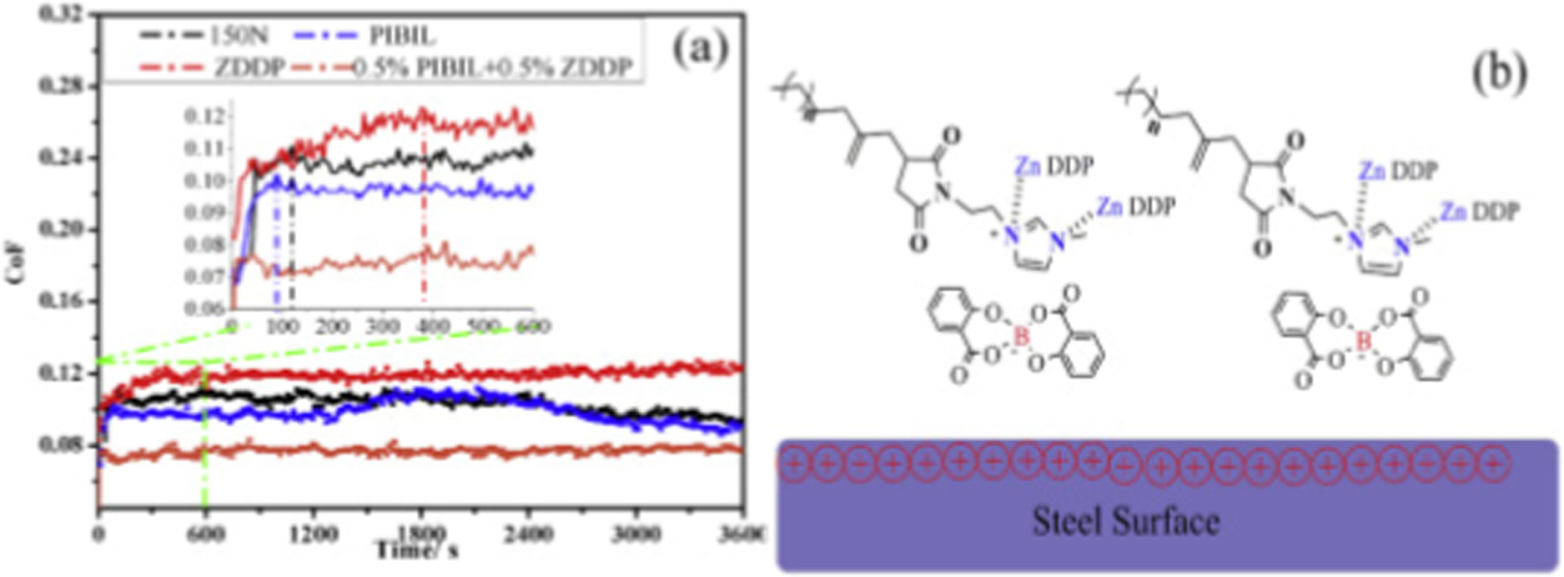
Coefficient of friction as a function of time (a) and complex formed by ZDDP and PIBIL (b). Adapted with permission from [ref 46]. Copyright [2017]­[Elsevier].

Finally, the effectiveness of PIBIL was also demonstrated in fully formulated engine oil. The addition of a small amount of PIBIL to a commercial SAE 5W-30 lubricant resulted in measurable reductions in both friction and wear, along with smoother and more stable tribological behavior. These improvements closely mirror the synergistic effects observed in base oil formulations, indicating that PIBIL can integrate effectively with existing additive packages, including ZDDP, without compromising the formulation stability.

### Poly­(Ionic Liquid)­s Dispersants: Structure and Function

PILs are a unique subclass of polyelectrolytes in which each repeating unit of the polymer backbone carries an IL moiety.^67,68^ Unlike conventional polyelectrolytes, the ionic centers in PILs are covalently tethered to the polymer chain, conferring a distinct combination of IL functionality with macromolecular stability.^69^ While ILs are typically liquids at room temperature, PILs are generally solid, yet they retain low glass transition temperatures compared to conventional ionic glasses, thereby enhancing their flexibility and processability.^70^

The structure–property correlations of certain types of ILs are tabulated in Table 2. The major advantages of PILs over ILs include improved mechanical robustness, enhanced durability, and better spatial organization of ionic domains, while still retaining the ionic conductivity and tunable physicochemical characteristics inherent to ILs. Their resistance to thermal degradation is likewise preserved, allowing PILs to operate effectively under elevated temperatures.^67,71^ Depending on the choice of cation–anion pair, PILs can be designed with imidazolium, pyrrolidinium, or phosphonium backbones, each offering tailored solubility, interfacial activity, and dispersibility in complex matrices such as lubricants.^72,73^ This molecular versatility allows PILs to function as highly effective dispersants and stabilizers for soot and sludge in engine oils, mitigating aggregation, and enhancing lubricant efficiency under severe operating conditions.

**Table 2:** Structure and Properties of ILs

ionic liquids	structure	properties	uses	refs
imidazolium-based ILs	imidazolium ring (C_4_H_4_N_2_) with alkyl chains attached at positions 1 and 3	high thermal stability	high ionicity and low volatility leading to better stability in high performance engine oils and decreasing evaporative losses	^74,75^
	example: 1-butyl-3-methylimidazolium hexafluorophosphate	low viscosity		
		can dissolve a wide variety of organic and inorganic compounds		
		high ionic conductivity and low volatility		
phosphonium-based ILs	phosphonium cations [PR_4_]^+^ with long alkyl chains	good thermal stability	potential lubricants, with the ability to reduce friction and wear depending on the alkyl chain length	^76,77^
	example: trihexyltetradecyl phosphonium chloride	higher polarity		
		good solubility		
		low volatility		
ammonium-based ILs	quaternary ammonium salts [R_4_N]^+^ with alkyl groups	high ionic conductivity and stability at elevated temperatures	A quaternary ammonium with four butyl groups, making it hydrophobic and highly soluble in oils and nonpolar solvents	^59,78^
	example: tetrabutylammonium bis(trifluoromethylsulfonyl) imide			
pyrrolidinium-based ILs	Pyrrolidinium cation [C_4_H_9_N]^+^ with alkyl chains	good thermal stability	Its potential in applications such as extreme pressure lubrication due to its unique properties	^62,79^
	example, *N*-butyl-*N*-methylpyrrolidinium bis (trifluoromethanasoulfonyl) imide	low viscosity		
		wide electrochemical stability window		
guanidium-based ILs	guanidinium cation [C(NH_2_)]_3_^+^ with alkyl chains	high thermal stability	demonstrating excellent antiwear and friction-reducing properties	^63,80^
	example: guanidinium bis (trifluoromethylsulfonyl) imide	good electrochemical stability		

Beyond tribological applications, PILs have also been investigated as solid ionic conductors for batteries and fuel cells,^72^ as precursors for porous carbons and catalytic materials,^70,71^ and as stabilizers in advanced colloidal systems.^73^ This convergence of IL chemistry and polymer design places PILs at the forefront of multifunctional material development with significant promise for next-generation lubricant formulations that combine stability, dispersibility, and environmental compatibility.

### Characterization Techniques for PIL and IL-Based Dispersants

#### Surface Tension and Interfacial Properties

The surface tension and interfacial properties of PIL dispersants provide fundamental insight into their functional roles in lubricants. These interfacial characteristics directly influence lubrication efficiency, colloidal stability, and the ability of the dispersant to interact with metallic surfaces under high shear conditions.^38^ Due to its amphiphilic nature, PIL dispersants lower the surface tension of the base oil matrix, thereby enhancing spreading and wetting on engine surfaces.^81^ Improved wettability facilitates the formation of stable lubricating films, which are critical for minimizing friction, mitigating wear, and sustaining long-term lubrication performance under dynamic operating environments.^82^ Given that lubricants are exposed to fluctuations in temperature, shear rate, and contaminant load, the ability of PIL dispersants to maintain interfacial stability under such conditions is a key determinant of oil endurance and reliability.^83^ The surface tension in PIL-containing lubricant systems is commonly achieved by using advanced techniques such as drop shape analysis, maximum bubble pressure, pendant drop, and Wilhelmy plate methods. These techniques provide precise quantification of surface tension, including its dependency on concentration and temperature.^84,85^ These methods allow for direct monitoring of interfacial changes induced by PIL dispersants. Additionally, the Langmuir–Blodgett (LB) technique offers structural insights into the molecular packing and film-forming capability of PILs at the oil–air or oil–metal interface, highlighting their ability to organize into stable interfacial layers that reinforce antiwear performance.^86^

Previous works have consistently reported that PILs substantially reduce the surface tension of oils, thus improving lubricant spread ability and surface coverage, which translates to lower frictional losses.^50^ Imidazolium- and phosphonium-based PIL dispersants have been shown to decrease the surface tension of hydrocarbon base oils from ∼35 mN m^–1^ to ∼20 mN m^–1^ at concentrations near their critical micelle concentration (CMC), indicating strong surface activity and micelle formation.^87,88^ This reduction in surface tension not only enhances wetting on metallic substrates but also promotes the formation of ordered interfacial films capable of withstanding high shear stress, thereby minimizing asperities contact.^86^ Importantly, the degree of surface tension depression is highly dependent on the cation–anion pairing of PILs, where longer alkyl-substituted cations and bulky, weakly coordinating anions (NTf_2_^–^, PF_6_^–^) exhibit stronger amphiphilic behavior and more pronounced interfacial activity.^89,90^ Temperature is a crucial factor, as surface tension measurements indicate that PIL-modified oils maintain reduced interfacial energy even at elevated temperatures (>100 °C), suggesting that dispersant activity is preserved under engine-relevant conditions.^71^ These findings confirm that the surface tension-lowering effect of PILs is not only a physicochemical indicator of surface activity but also provides insights for tribological efficiency, since stable, low-energy interfaces directly translate into reduced boundary friction, improved antiwear performance, and extended lubricant lifetime.

The CMC and corresponding surface tension behavior of PIL dispersants are strongly governed by the molecular structure of the IL moiety, particularly the cationic headgroup, alkyl chain length, and counteranion. Longer hydrophobic alkyl substituents on the phosphonium cation enhance amphiphilicity and reduce the CMC, as stronger hydrophobic interactions promote earlier micellization and tighter packing at the oil–air interface.^91^ Similarly, bulky or weakly coordinating anions, such as bis­(trifluoromethanesulfonyl)­imide ([NTf_2_]^−^), lower surface tension more effectively compared to halide anions, due to their ability to facilitate ion-pair dissociation and increase interfacial mobility.^87^ In contrast, shorter alkyl chains or highly hydrophilic counterions raise the CMC and limit the surface tension reduction, as weaker hydrophobic driving forces hinder micellar aggregation.^88^ These trends are consistent with PILs, where the incorporation of long polyisobutylene tails coupled with flexible phosphonium headgroups enables efficient interfacial adsorption and pronounced lowering of the surface tension until the CMC is reached.^90^ Such structure-CMC correlations not only define the concentration-dependent interfacial activity of PIL but also dictate their lubrication efficiency by optimizing surface coverage and film formation on metallic substrates under high shear conditions. The CMC not only reflects the self-assembly threshold of PIL dispersants but also serves as a performance indicator for lubrication and dispersion efficiency. At concentrations below the CMC, PIL molecules adsorb at the oil–air or oil–metal interface, progressively lowering surface tension and enhancing wettability, which promotes the uniform spreading of lubricant films across metal surfaces.^92^ Once the CMC is reached, the interface becomes saturated with PIL, ensuring maximum reduction in surface tension and optimal surface coverage for antiwear protection. Beyond the CMC, excess dispersant molecules aggregate into micelles in the bulk phase rather than contribute further to interfacial stabilization. This balance is critical: dispersant concentrations close to but not far beyond the CMC maximize both film stability and colloidal dispersion of contaminants.^88^ A well-defined CMC therefore, enables precise dosing of PIL in lubricants, preventing overdosing, which may increase viscosity, reduce oil flow, or destabilize colloidal suspensions, while ensuring sufficient surface-active molecules are present to protect engine components under shear and thermal stress.^87^ Thus, CMC determination through SFT analysis directly translates into an optimized operational window for dispersant performance, ensuring durability, efficiency, and stability in lubricant formulations.

#### Scattering Techniques

Scattering techniques are essential tools for probing the nanostructural organization and dynamic behavior of dispersant systems on the molecular and colloidal scale. In the context of lubricants, small-angle neutron scattering (SANS) and small-angle X-ray scattering (SAXS) provide critical insight into the size, shape, and spatial distribution of aggregates formed by PIL dispersants in oil matrices.^93,94^ These methods enable the direct observation of micellization, molecular packing, and structural evolution of PIL as a function of concentration, temperature, and shear stress, thereby complementing surface tension studies that identify CMC thresholds.^88^ SANS and SAXS analyses reveal whether PIL molecules exist as isolated entities, form elongated aggregates or develop into well-defined micellar structures under lubrication-relevant conditions.^87^ Such nanostructural information is pivotal in understanding how PIL dispersants stabilize soot and sludge particles, prevent aggregation, and maintain oil cleanliness. Furthermore, correlating scattering-derived parameters such as radius of gyration, Porod exponent, and fractal dimension with tribological performance allows linking interfacial activity to bulk structural behavior, establishing a molecular-to-macroscale relationship essential for rational design of next-generation PIL-based lubricant additives.

#### SANS

SANS provides unique structural insights into PIL dispersants in lubricants, particularly in the critical nanometer regime (∼1–10 nm) where aggregation, micellization, and interfacial self-assembly dominate performance.^95^ Unlike bulk techniques, SANS resolves the internal architecture of dispersant aggregates, including radius of gyration *R*_g_, correlation length, and fractal dimension, parameters that dictate colloidal stability and rheological behavior.^96^ For PIL dispersants, the balance between ionic headgroups and hydrophobic PIB tails governs their tendency to form discrete micelles, elongated cylindrical aggregates, or percolating networks. Each morphology correlates with distinct lubrication functions: spherical micelles improve soot encapsulation, worm-like structures enhance viscosity stabilization, and network-like assemblies provide antisedimentation reinforcement under shear.^72^ Contrast variation in SANS is achieved by selective deuteration of oil or dispersant segments, enabling domain-specific structural resolution. This is particularly advantageous for PILs, as it differentiates ionic headgroup solvation shells from the hydrocarbon-rich PIB backbone. Such nanoscale mapping allows one to monitor structural evolution across concentration regimes, for example, confirming micellization at the CMC derived from surface tension isotherms and capturing subsequent transitions into higher-order assemblies. SANS can be used to follow changes in aggregate structure with concentration. Using appropriate scattering models, such as the Guinier-Porod approach, transitions between different aggregation states such as unimers, spherical micelles, and higher-order assemblies can be identified from the scattering profiles.^97^ Importantly, these structural transformations directly influence dispersant performance: a well-defined CMC optimizes surface coverage and particle dispersion, while excessive aggregation beyond this threshold can induce penalties or reduce oxidative stability.^73,98^

While SANS data for PIL dispersants are still limited at present, the structural evolution of PIL dispersants can be directly linked and understood to SANS intensity profiles for other amphiphilic materials such as surfactants and detergents. The SANS data for alkyl acrylate copolymers is depicted in Figure 7.^99^ In the copolymer system, increasing the alkyl acrylate block length enhances segregation and drives stronger core formation, analogous to how longer alkyl substituents in PIL cations promote aggregation and the transition from discrete ionic clusters to extended domains. Similarly, the poly­(acrylate) block that stabilizes the micelle corona through electrostatic and steric repulsion parallels the ionic corona in PILs, where the charge density and counterion distribution govern aggregate stability. Variations in the degree of neutralization (α) mirror ion-pairing strength in PILs, with reduced charge repulsion in copolymers and stronger ion association in PILs both leading to compact, more aggregated morphologies. Furthermore, concentration-dependent scattering signatures in both systems reflect a shift from isolated aggregates to correlated clusters and ultimately to network-like domains, which critically influences viscosity and dispersion stability. Maeda et al.^100^ also reported such transitions of amphiphiles in oil, where micelle radii and aggregation number were extracted via model fitting. In another study, SANS data for IL mixtures exhibited clear structure factor modulation consistent with micellization and microphase separation in [C_nmim]­[Tf_2_N] systems, emphasizing how alkyl chain length impacts aggregation morphology.^101^ Additionally, SANS investigations of imidazolium-based ILs revealed transitions from spherical micelles to bicontinuous microemulsions at elevated concentrations. This information directly translatable to PIL-based systems used for soot and sludge dispersion.^102^

**Figure 7:**
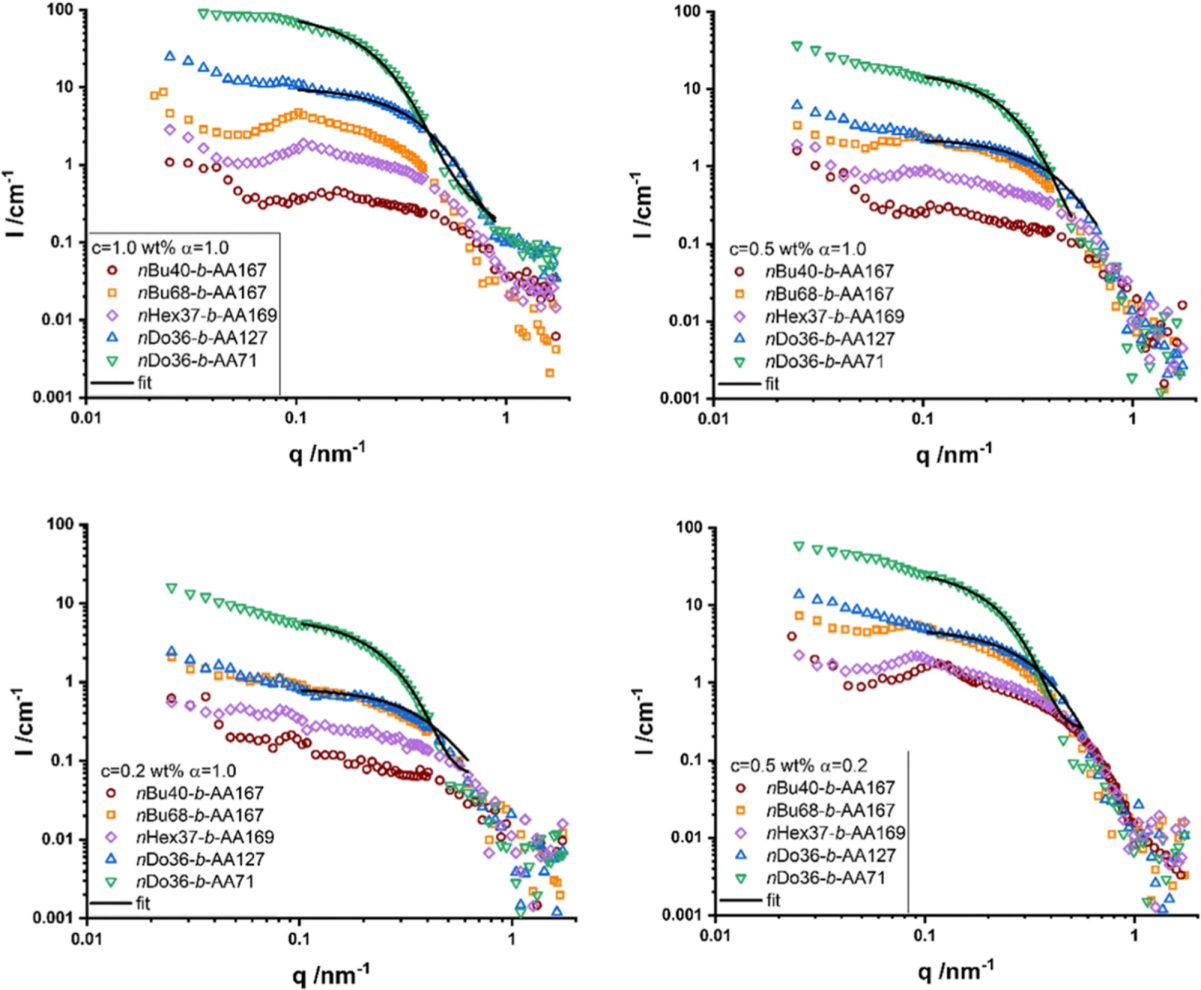
SANS intensity as a function of q for poly­(acrylate) block samples with 0.2 to 1.0 wt % at α = 1.0, and for 0.5 wt % also for α = 0.2 with fit curves for Guinier regime. Adapted with permission from [ref 99]. Copyright [2020]­[Springer Nature].

Coupling SANS-derived insights into nanoscale structural transitions with surface tension-based determination of CMC provides a powerful framework for understanding the self-assembly and interfacial activity of PIL dispersants. This integrated approach allows for the fine-tuning of dispersant dosage to achieve optimal interfacial coverage, stable particle dispersion, and controlled micellization, while avoiding excessive aggregation that may compromise oil rheology, oxidative resistance, or additive compatibility.^103^ Such a multiscale mechanistic perspective bridges molecular structure with macroscopic performance, guiding the rational design of next-generation PIL dispersants capable of delivering enhanced lubrication, thermal stability, and long-term reliability in advanced engine systems. This multiscale mechanistic understanding links molecular architecture to macroscopic behavior, supporting the rational development of next-generation PIL dispersants that provide improved lubrication, resistance to thermal degradation, and long-term reliability in advanced engine systems.

#### SAXS

SAXS is a powerful structural characterization technique widely applied to investigate nanoscale organization in complex materials, including PIL dispersants in lubricating oil formulations.^104,105^ Similar in principle to SANS, SAXS operates in the low-Q region to resolve structures in the ∼sub 100 nm range but employs X-ray photons rather than neutrons as the scattering probe. This distinction makes SAXS particularly advantageous in cases where contrast between phases depends primarily on electron density differences, such as when isotopic variation is not practical or when high-resolution structural data from electron-rich elements are required.^95^ In lubricant additives, SAXS offers quantitative insight into self-assembly phenomena of the dispersants, including micellization, worm-like micelle formation, and lamellar domain development, as well as their size–concentration relationship. Such measurements are critical to understanding aggregation dynamics and their influence on the rheological properties, phase stability, and dispersant efficiency. By resolving the internal arrangement of small aggregates, SAXS directly links nanoscale morphology to macroscopic tribological performance, such as friction reduction, wear prevention, and soot suspension capacity in high-stress lubrication environments.^106^

Zare et al. (2012)^107^ used SAXS to examine the nanoscale morphology of PIB-based IL dispersants, showing how variations in ionic headgroup chemistry influence their structural organization and resistance to thermal degradation. A series of PIB-ILs with methylimidazolium, pyrrolidinium, or triethylammonium end groups were examined, revealing well-defined Bragg reflections indicative of ordered nanostructures. Methylimidazolium-terminated PIB-ILs exhibited highly stable morphologies with minimal thermal disruption up to decomposition, whereas pyrrolidinium and triethylammonium substitutions underwent temperature-dependent order–order and disorder–order transitions typical of ionomers. These SAXS-derived structural features were directly reflected in the macroscopic behavior, where persistent ordering improved viscoelastic response and regulated flowability. The thermal robustness of the dispersants could also be adjusted by tailoring the ionic headgroup chemistry. György and co-workers^108^ employed SAXS to characterize PLMA–PMMA copolymer nanoparticles in mineral oil, determining the micellar sizes and aggregation numbers via fitted form-factor models as depicted in Figure 8. From the figure, the dispersant having a worm-like structure of an aggregation number of 190 with a micellar size of 14 nm, when about 1.0 wt % of the PLMA–PMMA was added. Chen and Evans^109^ applied in situ SAXS to monitor self-assembly of imidazolium-based PIL dispersants in synthetic ester base oils under elevated temperature, observing a thermally induced transition from spherical micelles to elongated rod-like aggregates above 80 °C, directly correlating morphological transitions to changes in viscosity and dispersant performance. These findings highlight the capability of SAXS to resolve nanoscale structural features relevant to the dispersant performance in lubricant formulations.

**Figure 8:**
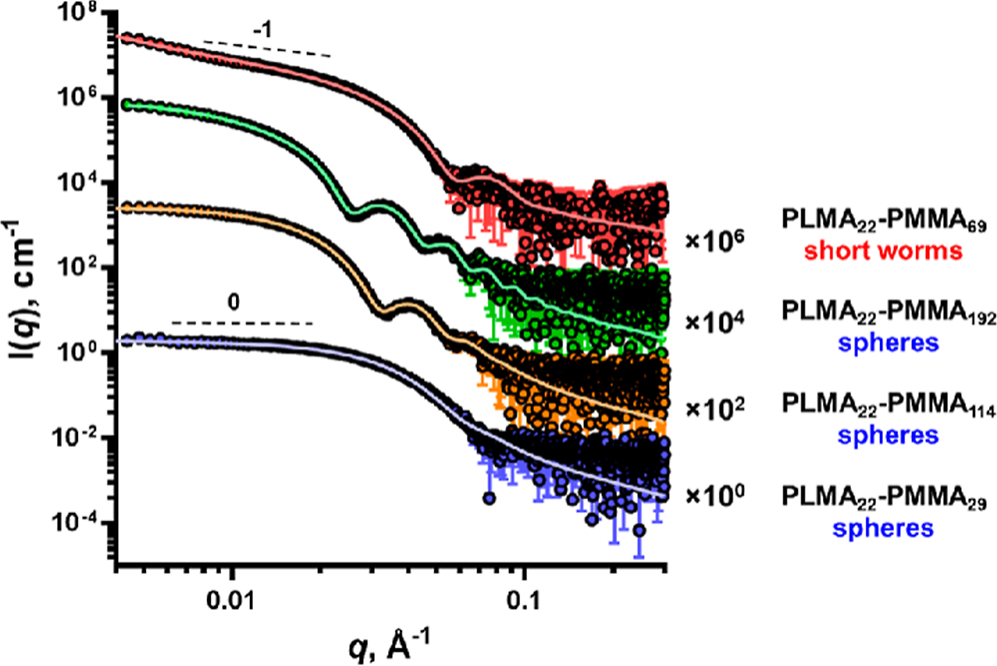
Structure-dependency of PLMA–PMMA in mineral oil analyzed from SAXS. Adapted with permission from [ref 108]. Copyright [2021]­[ACS].

#### Zeta-Potential Analysis

Zeta potential serves as a vital diagnostic for gauging the electrostatic stabilization of IL-based dispersants across diverse fluid systems including lubrication settings. Khan and co-workers (2020)^110^ reported that bis-phosphonium ILs (2P888–C4) formed stable oil-in-water emulsions whereby positive zeta-potential values indicated the cationic layer predominated at the droplet interface, thus promoting the enhancement of emulsion stability and significantly reducing wear and friction in steel tribosystems. In nanolubricant systems. Ismail and co-workers^111^ established threshold values for colloidal stability, with the zeta-potential values above ±45 mV regime, corresponding with excellent lubrication performance in TiO_2_/polyvinyl ether-based oils, whereas values below ±15 mV correlated with unstable, sediment-prone dispersions. Relevance to this context, the effective lubrication of PIB–IL dispersants suggests that targeting a zeta-potential value of at least 40–50 mV would enhance long-term colloidal stability and mitigate sludge formation, especially in low-polarity base oils.

While electrostatic stabilization is weakened in nonpolar media, studies on surface charge influenced by the polymeric dispersants remain a challenge due to its system complexity. In nonpolar media, where the Bjerrum length is typically low, the separation at which electrostatic interaction energy equals thermal energy is extremely short (typically <1 nm) due to the low dielectric constant of the medium (ε ≈ 2–5).^112^ This short Bjerrum length weakens long-range electrostatic interactions, meaning that zeta-potential values in oils are intrinsically lower than those in aqueous systems for the same surface charge density. Consequently, for PIBIL dispersants, colloidal stability does not solely rely on electrical double-layer repulsion, particularly in low-permittivity media such as mineral oils, where the Bjerrum length is large (≈28–30 nm) and the effective electrostatic screening length is severely limited.^113^ Instead, a synergistic stabilization mechanism is required, combining steric hindrance from the PIB tail segments with localized, short-range electrostatic repulsion generated by the IL head groups. For example, polyisobutylene-functionalized trihexyltetradecylphosphonium decanoate (PIB–[P_6_,_6_,_6_,_14_]­[Dec]) has demonstrated enhanced nanoparticle dispersion stability in base oils, where small but measurable increases in the zeta-potential value, from +8 mV to +18 mV, were sufficient to suppress aggregation over extended aging tests due to the concurrent steric shielding provided by the PIB chains.^16^ Similarly, PIB–imidazolium ILs such as PIB-–[C_8_mim]­[BF_4_] exhibited stable TiO_2_ and SiO_2_ nano dispersions in PAO-6, with zeta potentials below the conventional ±30 mV “stability threshold,” yet no significant sedimentation was observed over 90 days owing to strong solvation and brush-like PIB corona formation.^38^ In another case, PIL dispersants based on ammonium and phosphonium ILs achieved long-term graphene nanoplatelet stability in synthetic esters, where zeta potentials remained in the +12 to +20 mV range but performance in tribological tests showed significant wear scar diameter reduction, confirming that steric–electrostatic synergy can compensate for weak electrical double-layer effects in nonpolar environments.^114^ These findings demonstrate that zeta-potential analysis remains a valuable tool for ionic-liquid-based dispersants in lubricating oils, as even modest positive shifts in the absolute values, together with steric stabilization, can strongly affect colloidal stability, dispersant efficiency, and tribological performance under operating conditions.

### Application in Lubrication

#### Engine Oils Lubricants

The performance of dispersants in engine oils is inherently governed by their nanoscale structural organization and interfacial properties. Soot stabilization requires dispersants that not only prevent particle agglomeration through steric and electrostatic repulsion but also sustain a stable dispersion under the elevated temperatures and shear conditions typical of engines.^115^ PILs dispersants offer unique advantages, as their amphiphilic architectures promote micelle-like aggregation in oils, where the hydrophobic alkyl domains drive association while the ionic corona provides a tunable surface charge that ensures effective particle repulsion.^38,109^ Structural analysis via small-angle scattering demonstrates that longer alkyl substituents enhance core segregation, whereas higher ionic charge densities increase corona stability, both factors directly correlating with improved soot dispersion and reduced coagulation. Moreover, the ionic surface layer formed by PILs at metal interfaces reduces friction and wear, functioning as a protective boundary film even in highly contaminated environments.^116^ The balance between surface charge, counterion mobility, and aggregation state is thus central to their dual role: maintaining soot particles in suspension and providing durable antiwear protection. By integrating nanoscale structural control with surface electrostatics, PIL-based dispersants achieve superior oxidative resistance, extended oil lifetimes, and lower soot-induced viscosity growth compared to conventional succinimide-based systems, thereby offering both improved engine efficiency and reduced environmental footprint.^4,117^

#### Industrial Lubricants

PIL dispersants hold transformative potential across a spectrum of industrial lubrication systems beyond engine oils owing to their unique nanoscale aggregation and tunable surface charge properties. In gear oils, PILs form resilient ionic boundary films on metallic surfaces, enhancing scuffing resistance and load-bearing capacity, thereby extending equipment lifetimes.^114,118^ Hydraulic systems benefit from the PILs’ amphiphilic architecture, which supports stabilization of wear debris and contaminants, reduces sludge formation, and promotes consistent pump performance under cyclic pressures and temperatures.^90^ In metalworking fluids, PILs dual-function, hydrophobic domains improve lubricity while ionic characters inhibit corrosion and minimize adhesion between tools and workpieces, significantly decreasing tool wear and improving surface finishes.^80^

Marine and offshore systems, characterized by corrosive saline conditions and thermal extremes, find PILs particularly effective: their electrostatic surface interactions and thermal robustness mitigate oxidation and corrosion, meeting stringent environmental performance standards.^117^ Blended into grease formulations, PILs also excel as dispersants for solid lubricants such as MoS_2_ or graphite, preventing aggregation and ensuring uniform distribution for dependable lubrication under elevated temperatures and pressures.^116^ Further, broader literature on ILs and PILs underlines their efficacy as green corrosion inhibitors: they adsorb onto metal surfaces such as steel and aluminum, forming protective films that inhibit oxidation and chemical degradation with high thermal and chemical stability. These diverse applications collectively demonstrate that PILs, through their tunable nanoscale aggregation, ionic corona architecture, and strong interfacial adsorption, function as next-generation dispersants capable of delivering improved soot and debris stabilization, robust antiwear and anticorrosion protection, and extended lubricant lifetimes. By simultaneously enhancing energy efficiency, mechanical reliability, and environmental sustainability, PIL-based dispersants provide a versatile platform for advancing industrial lubrication technologies across both automotive and nonautomotive sectors.

## Conclusion

The diverse structural architectures of PILs, spanning backbone composition, side-chain length, and counterion chemistry, are central to their aggregation, interfacial activity, and self-assembly behavior. These characteristics directly determine their performance in lubrication, dispersion stabilization, and related industrial functions. Current progress has been enabled by complementary characterization approaches; surface tension analysis reveals adsorption and wettability; SAXS and SANS capture nanoscale ordering and structural evolution; and zeta potential provides direct insight into electrostatic stabilization and colloidal interactions. Collectively, these techniques establish a multiscale perspective that links molecular structure to macroscopic functionality. Nevertheless, important challenges remain. Most existing studies address isolated aspects of PIL behavior, often under simplified conditions that do not fully replicate industrial environments. The coupling between ionic aggregation, dynamic restructuring under shear, and long-term stability remains insufficiently understood. Moreover, systematic frameworks that integrate SFT, SAXS, SANS, and electrokinetic measurements under operando conditions are still scarce. Future research should therefore emphasize multitechnique, time-resolved investigations to capture the dynamic evolution of PIL structures under realistic operating conditions. Advanced computational modeling, combined with experimental scattering and interfacial studies, will be pivotal in predicting structure–property relationships with greater precision. Expanding this knowledge base will accelerate the rational design of PILs with tunable architectures for next-generation lubricants, dispersants, and sustainable functional materials. By bridging structural chemistry, advanced characterization, and application-driven design, PILs can be positioned as transformative materials that address pressing demands for efficiency, durability, and environmental responsibility in modern industrial systems.

The technological advancements of IL and PIL dispersants will be an integrated approach that combines data-driven molecular design with a structural-function-based understanding. AI-enabled molecular generation and machine-learning predictive frameworks can accelerate the screening of cation–anion combinations and PIL backbone architectures for the rapid identification of structures with optimized polarity, interfacial affinity, and oxidative durability. Complementary simulations across multiple scales, including quantum chemical calculations, atomistic and molecular dynamics, and mesoscale aggregation modeling, are essential to elucidate ion–soot interaction pathways, adsorption energetics, and competitive degradation mechanisms under engine-relevant conditions. Real-time and in situ experimental techniques such as synchrotron SAXS and SANS, high-temperature AFM, and shear-coupled spectroscopy are essential to validate computational predictions and monitor the structural evolution of dispersants under thermal, mechanical, and oxidative stress. Moreover, the IL and PIL compatibility within low-SAPS lubricant systems and their synergistic interactions with detergents, antioxidants, and antiwear additives must be systematically evaluated for practical formulation. These research directions collectively pave the way for the rational development of next-generation IL and PIL dispersants, enabling sustained high-performance stability in advanced engine lubrication applications.
